# Successful treatment of endogenous endophthalmitis with extensive subretinal abscess: a case report

**DOI:** 10.1186/s12886-018-0908-x

**Published:** 2018-09-05

**Authors:** He Xu, Bo Fu, Chunguang Lu, Li Xu, Jing Sun

**Affiliations:** 1 Department of Ophthalmology, The 4th People’s Hospital of Shenyang, Shenyang, Liaoning People’s Republic of China; 2 Departments of Gastroenterology, The First Affiliated Hospital of China Medical University, Shenyang, Liaoning People’s Republic of China

**Keywords:** Endogenous endophthalmitis, Subretinal abscess, Vitrectomy, *Klebsiella pneumonia*, Treatment

## Abstract

**Background:**

Endogenous endophthalmitis could lead to a devastating outcome without a prompt and appropriate management. We report a case of advanced endogenous *Klebsiella pneumoniae* endophthalmitis with extensive subretinal abscess that was successfully treated with a vitrectomy.

**Case presentation:**

A systemically well 61-year-old man complained of ocular pain and visual decrease in his right eye for eighteen days. Ophthalmic examination showed dense inflammation in the anterior chamber and vitreous body. Systemic investigations discovered diabetes and no specific site of systemic infection was found after hospitalization. The inflammation continued to worsen after the intravitreal antibiotic injection. Therefore, a pars plana vitrectomy combined with phacoemulsification was performed. Intraoperatively, a white elevated, fluffy mass with the overlying retinal whitening and necrosis was revealed in superior periphery. In addition to this, extensive retinal hemorrhages and five adjacent subretinal whitish masses with exudative retinal detachment were observed in the posterior pole and inferior quadrants, which were suggestive of extensive subretinal abscess with intense overlying retinal inflammation. The excision of white fluffy mass superiorly was performed without retinotomy and aspiration of extensive subretinal abscess. The polymerase chain reaction of vitreous samples was positive for *Klebsiella pneumonia*. Intravitreal 2 mg/0.1 ml ceftazidime were repeated. Nine days after the surgery, the inflammation significantly subsided and the retina reattached. The patient was in a stable condition at subsequent visit eight months later.

**Conclusion:**

The delay in an accurate diagnosis and treatment caused extensive subretinal abscess combined with endogenous endophthalmitis. The treatment modality of subretinal abscess is typically individualized to the patient’s presentation. If the retina overlying the abscess is not necrotic, the extensive subretinal abscess can quickly absorbed after vitrectomy, retinotomy with aspiration of the abscess should be avoided to decrease the risk of retinal detachment.

## Background

Subretinal abscess is a particularly severe manifestation of endogenous endophthalmitis (EE), which can lead to a devastating outcome without timely and appropriate management. Various therapeutic approaches have been attempted with limited visual success. The present study examines a case of advanced EE combined with extensive subretinal abscess treated successfully with pars plana vitrectomy without retinotomy and aspiration of abscess. We present this case report for its unusual presentation and unprecedented recovery.

## Case presentation

A 61-year-old man presented with pain, redness, floaters and decreased vision in his right eye for two days. It was diagnosed as anterior uveitis at a local clinic and treated with prednisolone acetate eye drops combined with intravenous drip of dexamethasone and cephalosporin for seven days. Two weeks later, the patient’s visual acuity decreased to light perception, so he was referred to us eighteen days after his initial onset of symptoms. The patient had no previous history of systemic diseases or infectious diseases, no trauma or surgery before, no chronic medication used. He had a history of heavy drinking and chronic peptic discomfort, and he had been diagnosed suffering from peptic ulcer by agastroscopytwo weeks prior to the onset of symptoms. He had loss of appetite after abstinence from alcohol and reduced 15 kg of weight during the previous one month.

**Fig. 1:**
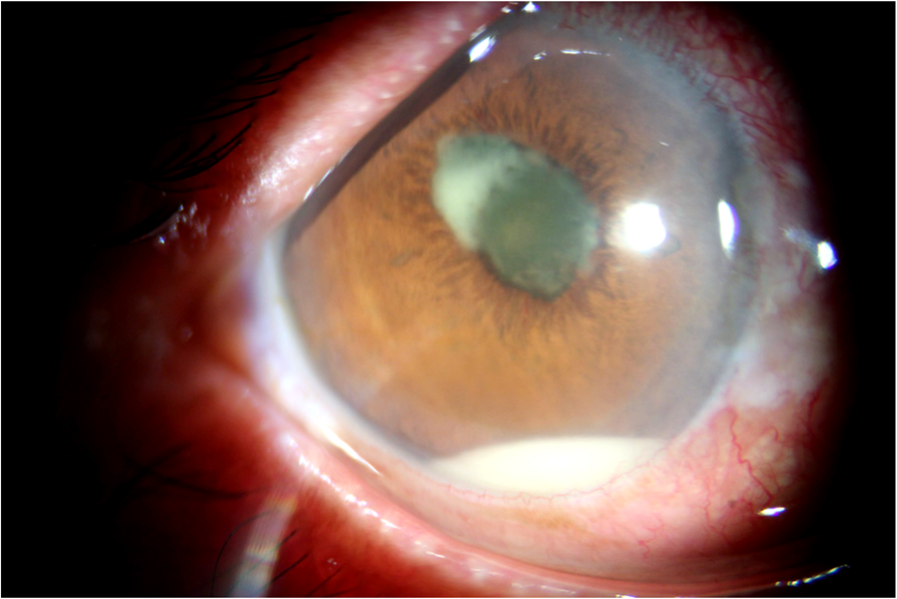
External photograph of right eye on presentation. Injected conjunctiva, a clear cornea, an approximately 1.4 mm hypopyon. Pupillary hypopyon, posterior iris synechia and fibrinous exudates covering the anterior lens capsule

**Fig. 2:**
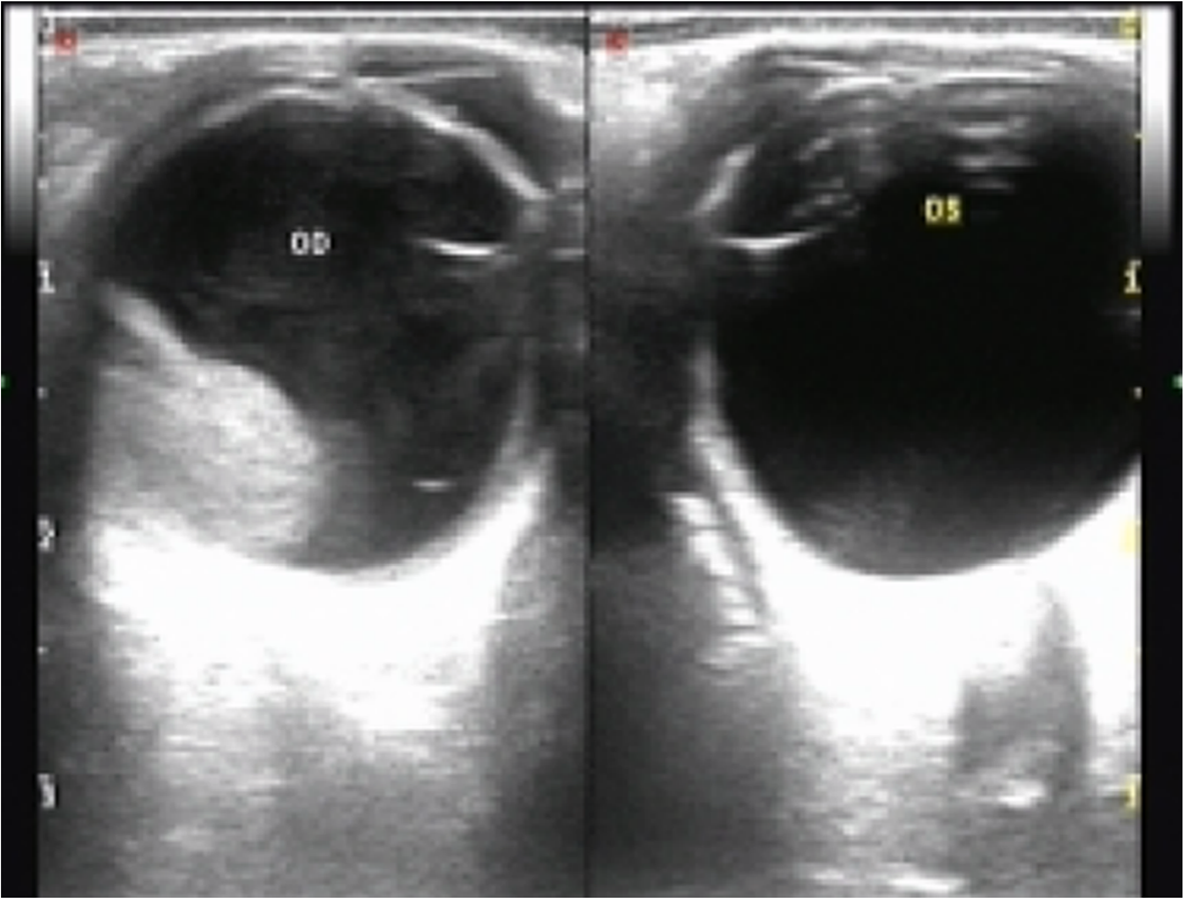
Color ultrasound examination of the eyeson presentation demonstrating dense vitreous opacities and avascular homogeneous hyperechoic mass in the right eye (OD), normal in the left (OS)

At presentation, vision was light perception in the right eye and 6/6 in the left. The slit-lamp examination of the right eye revealed mild injection, anterior chamber cells of 2+ with a hypopyon of 1.4 mm, pupillary hypopyon, posterior iris synechia, and fibrinous exudates covering the anterior lens capsule (Fig. 1). Fundus of the right eye was invisible due to the vitreous opacity and the left eye was normal. Intraocular pressure was normal in both the eyes. The color ultrasound examination revealed dense vitreous opacities and a avascular homogeneous hyperechoic mass (Fig. 2).

The vital parameters were in normal range, with the blood pressure of 132/80 mmHg, pulse 76/min and a temperature of 36.7° Celsius. Physical examination did not show any abnormalities. Systemic investigations including blood routine examination, liver and renal function tests, computed tomography scan of the lung and abdominal were all unremarkable. Serologic tests that included human immunodeficiency virus antibody, antibodies for toxoplasma, varicella zoster virus, herpes simplex virus and the treponemal antibody-absorption test yielded negative results. Laboratory result for fasting blood glucose was 9.07 mmol/l (normal range: 3.88~ 6.1). The patient was diagnosed as diabetes mellitus after many blood glucose tests and was treated with Metformin. No specific site of systemic infection was found. Presumptive diagnosis of right EE was made, but intraocular lymphoma could not be ruled out.

Vitreous and anterior chamber taps of the right eye were performed, aqueous and vitreous aspirations were sent for cultures and histopathology examination. The patient was treated with intravitreal injection of vancomycin 1 mg/0.1 ml and ceftazidime 2 mg/0.1 ml, topical levofloxacin 0.5% and prednisolone 1% acetate eye drops six times a day, atropine 1%ointment at night in the right eye, concurrent with intravenous drip of cefoperazone.

**Fig. 3:**
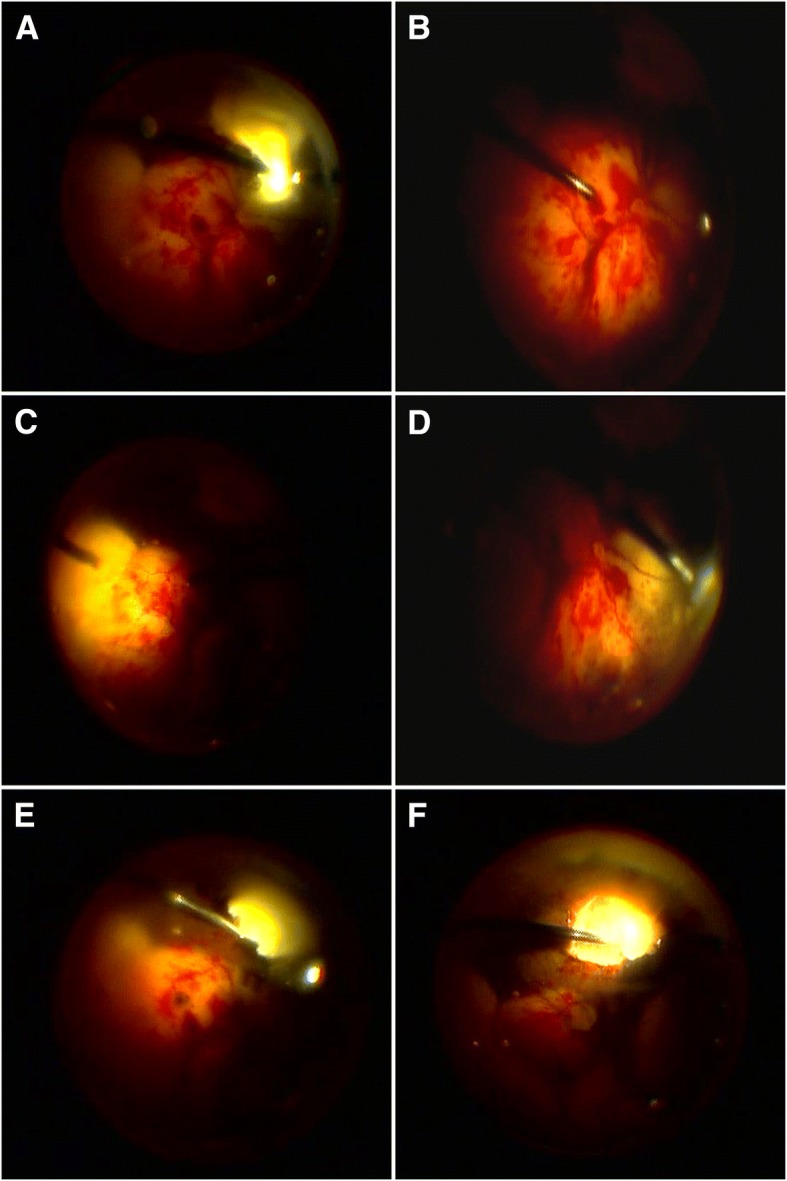
Intraoperative clinical findings. A white, elevated, fluffy mass with the overlying retinal whitening and necrosis was revealed in superior periphery, extensive retinal hemorrhages and 5 adjacent subretinal whitish masses with exudative retinal detachment in posterior polar and inferior quadrants. (**a**); The whitish subretinal mass in posterior polar. (**b**); in temporal quadrant (**c**); in inferior nasal quadrant (**d**). White fluffy cotton-like substance was excised from the superior mass (**e**). 4-disc diameter retinal defect left after the excision of the superior mass (**f**)

Three days after the intravitreal injection, the results of vitreous samples cultures and histopathology examination were all negative. The anterior chamber inflammation improved, and fundus of the right eye was still invisible. B-scan ultrasound showed increased vitreous debris and extensive thickening of the retina and choroid layer. Therefore, a pars plana vitrectomy (PPV) combined with phacoemulsification was performed. After a complete vitrectomy, a white elevated fluffy mass with the overlying retinal whitening and necrosis was revealed in superior periphery (Fig. 3a). In addition to this, extensive retinal hemorrhages and five adjacent subretinal whitish masses with exudative retinal detachment were observed in the posterior pole and inferior quadrants which were suggestive of extensive subretinal abscess with intense overlying retinal inflammation (Fig. 3b). Intraoperatively, we carefully cleared the white fluffy mass in superior and peripheral vitreous without retinal break formation. A white fluffy cotton-like substance was excised from the superior mass (Fig. 3c) and finally left a 4-disc diameter retinal defect (Fig. 3d). The vitreous and cotton-like substance were sent for culture, histopathology examination, and polymerase chain reaction (PCR) testing. Retinotomy and aspiration of extensive subretinal abscess in the posterior pole and inferior were not performed. Laser photocoagulation around the retinal defect site and gas or oil intraocular tamponade were not performed either. No intravitreal or intravenous antibiotics were used for we were not sure if the infection arose from fungal, bacterial, mycobacterial or a different aetiology. Post-operative administration remained topical levofloxacin 0.5%, prednisolone 1% acetate eye drops six times a day and atropine 1% ointment once a day.

**Fig. 4:**
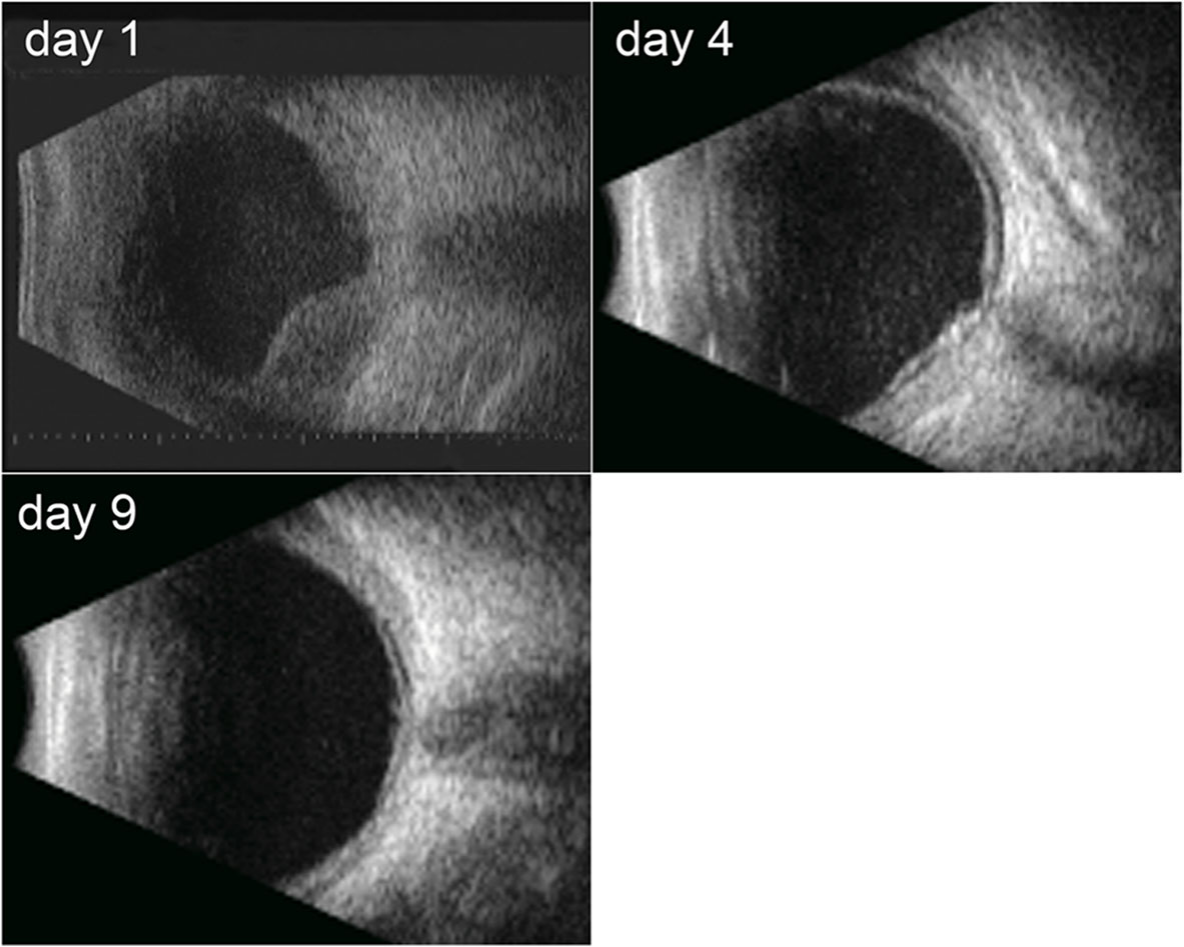
B-scan ultrasound of the right eye showing vitreous opacities and an extensive retinal detachment with sub-retinal exudates on postoperative day 1 (the upper left); slight vitreous opacities with shallow retinal detachment on postoperative day 4 (the upper right); the retina reattached except localized shallow retinal detachment on postoperative day 9 (the bottom left)

**Fig. 5:**
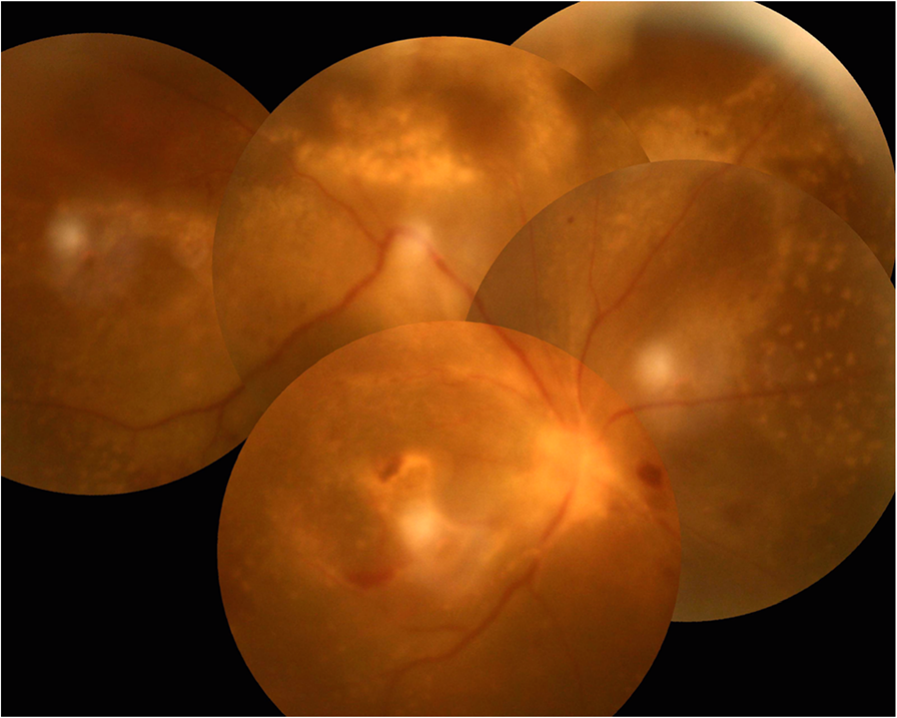
Fundus photograph on postoperative day 9. The retina reattached with some yellowish subretinal precipitatesand a scar in superior

**Fig. 6:**
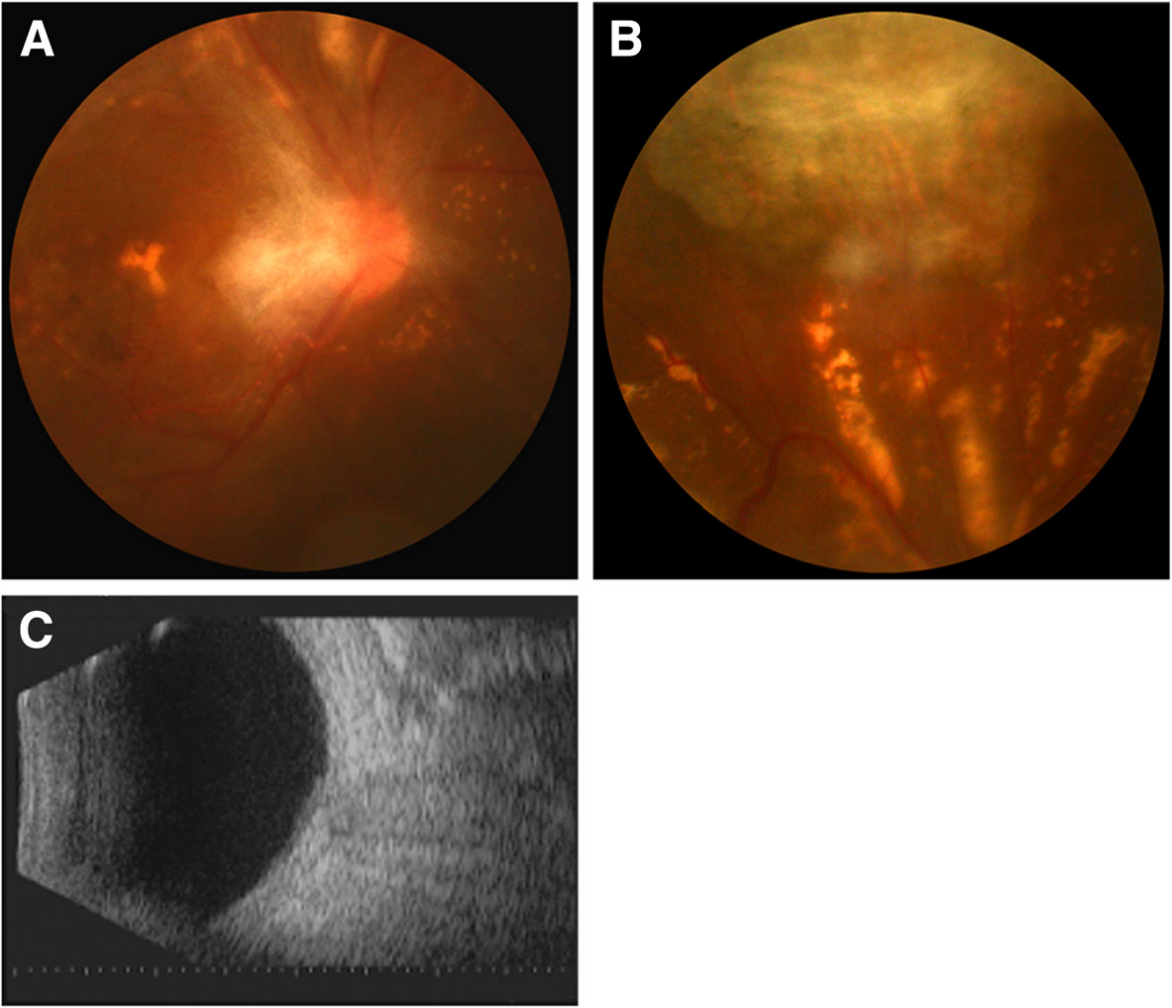
Fundus photograph and B-scan ultrasound of the right eye 2 months later. The retina attached with some yellowish subretinal precipitates (**a**), a large fibrotic scar superiorly (**b**), an epiretinal membrane in the posterior pole (**a**, **c**)

On post-operative day 1, slit-lamp examination showed anterior segment moderate inflammatory reaction and no posterior segment view because of vitreous opacities. B-scan ultrasound of the right eye showed vitreous opacities and an extensive retinal detachment with sub-retinal exudates (Fig. 4a). On post-operative day 4, the inflammatory reaction subsided significantly and the media started clearing. A blurry fundus was observed. B-scan ultrasound showed slight vitreous opacities and shallow retinal detachment (Fig. 4b). The result of vitreous samples PCR was positive for *Klebsiella pneumonia (KP)*. The results of cultures and histopathological examination were again negative. Clinical examination along with PCR testing confirmed the diagnosis of EE caused by *KP*. An intravitreal injection of ceftazidime 2 mg/0.1 ml on the right eye was performed. On post-operative day 9, the anterior chamber and vitreous cavity were clear, the retina reattached with lots of yellowish subretinal precipitates and a scar at the superior region (Fig. 5). B-scan ultrasound showed retina reattached except localized shallow retinal detachment (Fig. 4c). Corrected visual acuity improved to hand motions and intraocular pressure was normal. Intravitreal injection of ceftazidime 2 mg/0.1 ml was repeated in the right eye. The patient was discharged for follow-up as an outpatient with levofloxacin 0.5% and prednisolone 1% acetate eye drops administered topically for one week. He was in a stable condition at subsequent visit two months later. Fundus and B-scan ultrasound examination revealed the retina remained attached with some yellowish subretinal precipitates, a large fibrotic scar superiorly, an epiretinal membrane presented in the posterior pole (Fig. 6). At eight months, his eye remained quiescent with a corrected visual acuity of hand motions.

## Discussion

This is an unusual case of EEwith extensive subretinal abscess in a systemically well man. EE is a rare and severe disease with the potential to cause visual loss. The presentations of fundus with EE may be nonspecific such as vitritis, retinal hemorrhages, nerve fiber layer infarction, retinitis, perivasculitis, subretinal exudation and abscess. Subretinal abscess, a solitary and yellowish-white circumscribed lesion with hemorrhages in the overlying retina in the fundus, is an extremely rare presentation of EE. The treatment of subretinal abscess remains controversial and no standard management in the literature. Management options include intravenous antibiotics alone, PPV, PPV with intravitreal antibiotics, PPV with retinectomy or enucleation. As Eddie W. Harris and associates demonstrated, retinectomy with complete abscess excision and intravitreal antibiotic therapy could acquire relatively better visual recovery in EE [1]. Yet, the risk of retinal detachment after retinectomy and drainage of subretinal abscess was high [1–3], surgery for retinal detachment in these cases was difficult, and there was a need for long-term tamponade in such patients post vitrectomy [4]. Tsai TH and Peng KLconsidered that if the size of the subretinal abscess was smaller than four disc areas, PPV with intravitreal injection of antibiotics could be successful. If subretinal abscess was really large and high, vitrectomy combined with retinectomy to remove adequate abscess should be considered to decrease the amount of bacteria and to facilitate infiltration of antibiotics [5]. Recently, Venkatesh et al. had performed PPV along with injection of vancomycin directly into the subretinal abscess using a 41G needle in EE. Post-operative the abscess absorbed rapidly. Self-sealing small retinotomy created with a 41G translocation needle can be safely used to deliver drugs in a subretinal lesion decreasing the chance of proliferative vitreoretinopathy and retinal detachment [6].

Our patient had a delay in an accurate diagnosis and treatment, which caused the formation of extensive subretinal abscess. He received the PPV twenty-one days after initial symptoms appeared. Intraoperatively, the superior subretinal abscess was found to be broken with the overlying retinal necrotizing, and there were extensive subretinal abscesses with exudative retinal detachment located in posterior pole and inferiorly. So severe and extensive subretinal abscess involving in almost 90% of retina had not been reported. It was a dilemma whether to perform retinotomy and aspiration of the abscess or not, considering of the retina overlying the abscess presented with obvious inflammation and hemorrhage, retinotomy and photocoagulation in a fragile retina overlying the abscess was known to increase the risk of retinal detachment and proliferative vitreoretinopathy. Therefore no retinotomy with aspiration of the abscess and no endophotocoagulation were performed. We did not use intravitreal antibiotics and oil tamponade routinely in this case. To our surprise, extensive subretinal abscess was absorbed rapidly and retina reattached within nine days after vitrectomy. We infer that the vitrectomy removed necrotic tissue and most of the pathogenic bacteria and toxins in the eye, which resulted in quick resolution of acute inflammation. Retinal detachment did not occur although no endophotocoagulation around the retinal defect region resulted from excision of abscess in superior. We infer that the edge of necrotic retina and choroid lesions naturally formed adhesion, which achieved the goal of endophotocoagulative barrier. Although the prognosis of endogenous *KP* endophthalmitis was generally poor, especially with widespread subretinal abscess,in our patient, satisfactory anatomic outcome was achieved with vitrectomy and intravitreal ceftazidime.

Bacteria and fungi are the most common pathogens of EE. Culture and histological examination are commonly used to confirm the presence of a specific etiology. Another emerging technique is the use of PCR of aqueous and vitreous samples for detection of the etiology. In this case, vitreous samples were cultured twice anddid not grow any bacteria. PCR from the vitreous samples was positive for *KP*. PCR helped to identify the organism and confirmed the diagnosis of EE. Sowmya et al. demonstrated increased sensitivity of PCR over culture in their study [7]. PCR-based genetic assays technique has the advantage of rapid diagnosis, no fear of contamination of culture samples yielding false positive results and more sensitive than culture for identifying intraocular pathogens in endophthalmitis and uveitis, especially in samples that are culture negative [8, 9].

Our case was misdiagnosed as non-infectious uveitis in local hospital due to the absence of systemic infection. No foci of infection were identified in this case despite extensive investigation. We revealed some risk factors of immunocompromised, such as recently diagnosed diabetes, a history of heavy drinking, the gastroscopy examination two weeks prior to the onset of symptoms, peptic ulcer, and significantly loss of body weight. EE can be developed from the focus of infection in any part of the body as well as also may develop from normal flora after surgery as a result of hematogenous spread. In general, gram-negative organisms, especially KP are responsible for most cases of EE in East Asia [10, 11]. KP is a part of the healthy human microbiome, providing a potential reservoir for infection. KP can cause both localized and disseminated infections in various settings. Although it is not clear what started this patient’s disease process, it seems most likely that the gastroscopy examination lead to EE in the patient involving in immunocompromise states. EE after routine colonoscopy and dental cleaning had been reported [12–14], they considered that there was the theoretical risk of transient bacteremia resulting from any invasive procedure as endoscope because of the breakage of oral, gastral or intestinal mucosa.

## Conclusion

Our case is unique in that extensive subretinalabscess was successfully treated with a vitrectomy and aquired unprecedented recovery. Our experiences in this case demonstrate that the treatment modality of subretinal abscess is typically individualized to the patient’s presentation. If the retina overlying the abscess is necrotic, PPV with retinectomy to remove adequate abscess is required to decrease the amount of bacteria, whereas if the retina is not necrotic, retinotomy with aspiration of the abscess should be avoided to decrease the risk of retinal detachment and proliferative vitreoretinopathy, extensive subretinal abscess can be absorbed quickly.

## Abbreviations

EE: Endogenous endophthalmitis
*KP*: *Klebsiella pneumonia*
PCR: Polymerase chain reaction
PPV: Pars plana vitrectomy

## References

[1] Harris EW, D'Amico DJ, Bhisitkul R, Priebe GP, Petersen R (2000). Bacterial subretinal abscess: a case report and review of the literature. Am J Ophthalmol.

[2] Connell PP, O'Neill EC, Fabinyi D (2011). Endogenous endophthalmitis: 10-year experience at a tertiary referral Centre. Eye.

[3] Kaburaki T, Takamoto M, Araki F (2010). Endogenous Candida albicans infection causing subretinal abscess. Int Ophthalmol.

[4] Kitiratschky VB, Deuter C, Beck R (2015). Relationship between suspected reasons of intraocular inflammation and the results of diagnostic vitrectomy: an observational study. Ocul Immunol Inflamm.

[5] Tsai TH, Peng KL (2015). Metastatic endophthalmitis combined with subretinal abscess in a patient with diabetes mellitus-a case report. BMC Ophthalmol.

[6] Venkatesh P, Temkar S, Tripathy K, Chawla R (2016). Intralesional antibiotic injection using 41G needle for the management of subretinal abscess in endogenous endophthalmitis. Int J Retina Vitreous.

[7] Sowmya P, Madhavan HN. Diagnostic utility of polymerase chain reaction on intraocular specimens to establish the etiology of infectious endophthalmitis. Eur J Ophthalmol. 19(5):812–7. 10.1177/112067210901900520 19787602

[8] Okhravi N, Adamson P, Lightman S (2000). Use of PCR in endophthalmitis. Ocul Immunol Inflamm.

[9] Sandhu HS, Hajrasouliha A, Kaplan HJ, Wang W. Diagnostic utility of quantitative polymerase chain reaction versus culture in Endophthalmitis and uveitis. Ocul Immunol Inflamm. 2018:1–5. 10.1080/09273948.2018.1431291 29470930

[10] Wong JS, ChanTK LHM (2000). Endogenous bacterial endophthalmitis: an east Asian experience and a reappraisal of a severe ocular affliction. Ophthalmology.

[11] Ackson TL, Eykyn SJ, Graham EM (2003). Endogenous bacterialendophthalmitis:a 17-year prospective series and review of 267reported cases. Surv Ophthalmol.

[12] Wu AY, Oestreicher JH (2011). Endogenous bacterial endophthalmitis after routine colonoscopy. Can J Ophthalmol.

[13] Subramanian ML (2003). Topping TM. Endogenous endophthalmitis after routine dental cleaning. Arch Ophthalmol.

[14] Mali JO, Falk NS, Mali YP, Mencias L (2015). Endogenous endophthalmitis with iris abscess after routine dental cleaning. JAMA Ophthalmol.

